# Orocraniofacial findings of a Pediatric Patient with Joubert Syndrome

**DOI:** 10.5005/jp-journals-10005-1394

**Published:** 2016-12-05

**Authors:** Mridula Goswami, Anju S Rajwar, Mahesh Verma

**Affiliations:** 1 Professor and Head, Department of Pedodontics and Preventive Dentistry, Maulana Azad Institute of Dental Sciences, New Delhi, India; 2 Postgraduate Student, Department of Pedodontics and Preventive Dentistry, Maulana Azad Institute of Dental Sciences, New Delhi, India; 3 Director Principal and Head, Department of Prosthodontics, Maulana Azad Institute of Dental Sciences, New Delhi, India

**Keywords:** Hypoplasia, Magnetic resonance imaging, Molar tooth sign.

## Abstract

Joubert syndrome (JS) is a very rare autosomal recessive disorder, involving agenesis or dysgenesis of cerebellar vermis and brain stem. The neurological features of JS include hypotonia, ataxia, developmental delay, intellectual disability, abnormal eye movements, and neonatal breathing dysregulation. These may be associated with multiorgan involvement, mainly retinal dystrophy, nephronophthisis, hepatic fibrosis, and polydactyly. An obligatory hallmark feature associated with JS is the molar tooth sign (MTS), a complex midbrain-hindbrain malformation visible on brain imaging. This case report presents a pediatric case of JS in a 7-year-old girl. Joubert syndrome cases have been reported by various medical specialties in medical journals; however, this probably could be the first report of this rare developmental disorder in dental and oral health.

**How to cite this article:**

Goswami M, Rajwar AS, Verma M. Orocraniofacial findings of a Pediatric Patient with Joubert Syndrome. Int J Clin Pediatr Dent 2016;9(4):379-383.

## INTRODUCTION

Joubert syndrome (JS) is a very rare autosomal recessive condition, which was first described by the French pediatric neurologist Marie Joubert in 1969 in Montreal, Canada. It is characterized by episodes of abnormal respiratory pattern, oculomotor findings, hypotonia, ataxia, and developmental retardation with evidence of neuro-pathologic abnormalities of cerebellum and brainstem. Joubert syndrome is associated with the molar tooth sign (MTS), a radiologic finding that includes cerebellar vermis hypoplasia or dysplasia, thick and horizontally oriented superior cerebellar peduncles, and an abnormally deep interpeduncular fossa.^1^ Other malformations may also be present, such as extra fingers and toes, cleft lip or palate, tongue abnormalities, and seizures.

## CASE REPORT

A 7-year-old girl presented to the Outpatient Department of Pedodontics and Preventive Dentistry at Maulana Azad Institute of Dental Sciences, New Delhi, with the chief complaint of grossly decayed teeth and inability to eat. History revealed that the child was born with a birth weight of 3.4 kg by cesarean section and presented with breathing difficulties at birth and remained admitted to the neonatal intensive care unit for a period of 15 days. Mother of the patient reported that the child showed sluggish movements with little crying. The patient also presented with cleft of the palate and partial midline cleft-ing of the lip. The family history revealed that the patient had two sisters, aged 1 and 4 years, with no significant medical history.

**Fig. 1:**
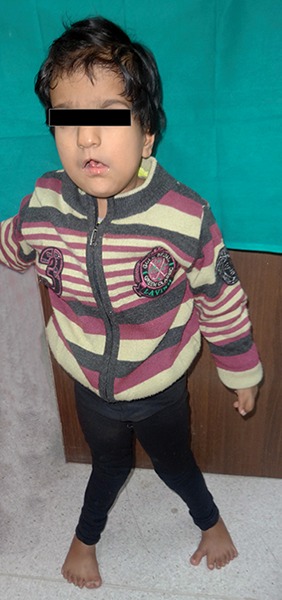
Ataxic gait of patient

**Fig. 2:**
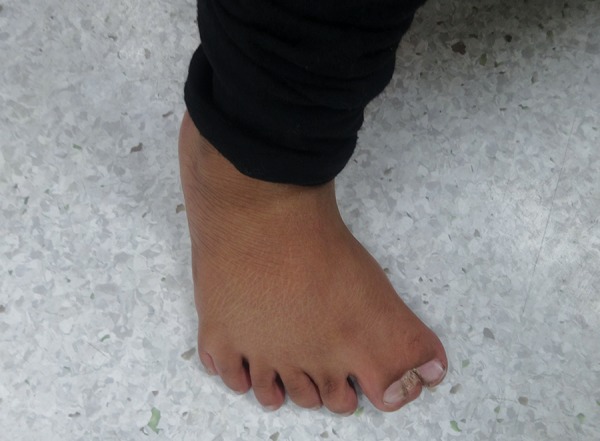
Polysyndactly of feet

**Fig. 3:**
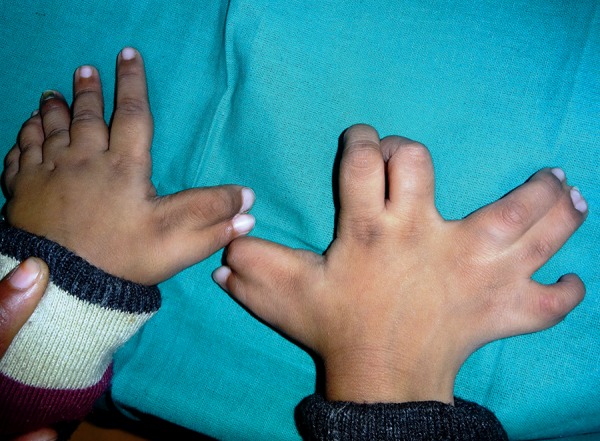
Polysyndactly of hands

On physical examination, it was observed that the vitals of the patient were normal. The child had an ataxic gait, with inward bowing of the legs, and walked with support. The fine motor skills examination revealed that there was no mature grip present (Fig. 1). The patient was observed to make occasional monosyllable sounds. The patient had flattened nasal bridge with the presence of hypertelorism and low-set ears. There was presence of syndactyly and Polydactyly of all the four limbs (Figs 2 and 3). Delayed milestones and mental retardation were Present.

**Fig. 4:**
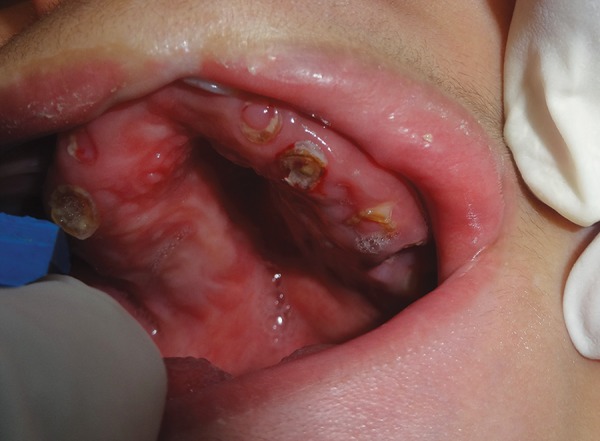
Intraoral view

### Dental Examination

There was Presence of cleft Palate and Partial midline clefting of liP along with enlarged tongue. There were eight teeth Present in the maxillary arch and seven teeth in the mandibular arch. The teeth were seen as multiPle root stumPs, which were grossly carious. There was significant gingival inflammation and enlargement along with excessive bleeding on Probing. The oral hygiene status of the patient was found to be very poor (Fig. 4).

**Fig. 5:**
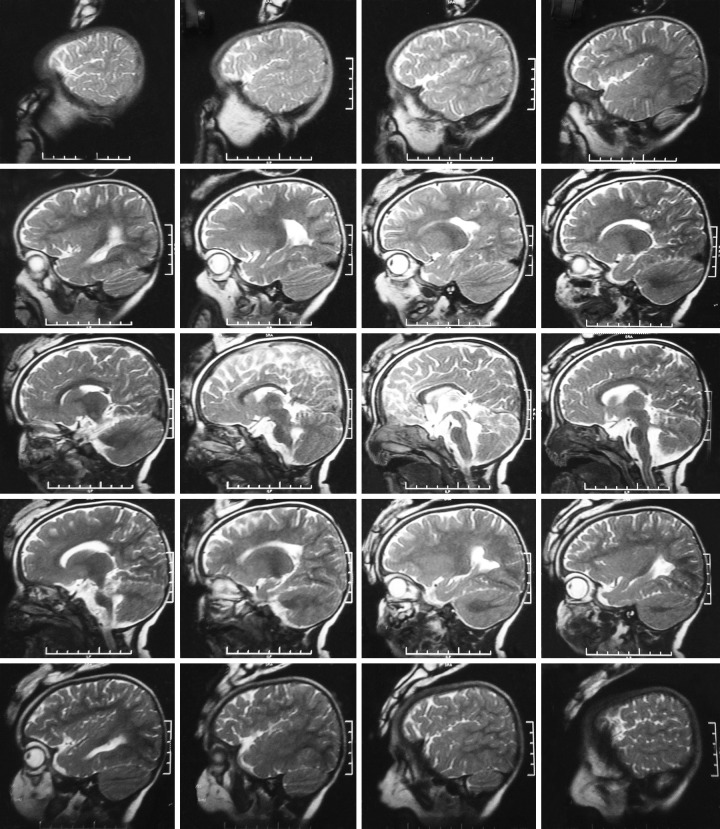
Computed tomography scan of brain

To diagnose JS, the Patient had undergone magnetic resonance imaging (MRI) scan. The axial T1-weighted and T2-weighted MRI showed hypoplastic vermis with nonfused cerebellar hemisphere and thin cerebrospinal fluid cleft between cerebellar hemispheres. The images also revealed hypoplasia of the brain stem and “molar tooth appearance” of the lower midbrain and upper pons. The 4th ventricle was enlarged and had a “bat wing appearance.” Normal signal intensity was seen in the cerebral parenchyma, basal ganglia, thalami, and brain stem. No hemorrhage was seen. The normal flow voids are maintained and the sulci, remaining ventricular systems, and basal cisterns were normal. The pituitary, orbits, and auditory meati were normal, and no midline shift was seen. Thus, MRI revealed hypoplastic vermis with nonfused cerebellar hemispheres with hypoplasia of the brain stem, and molar tooth appearance of the lower midbrain and upper pons, along with bat wing appearance of the 4th ventricle features, suggestive of congenital vermian hypoplasia JS (Fig. 5).

### DISCUSSION

Joubert syndrome was originally described in 1968 in four siblings with agenesis of the cerebellar vermis presenting with episodic hyperpnea, abnormal eye movements, ataxia, and intellectual disability.^1^ The incidence of JS has been estimated to be between 1/80,000 and/100,000 live births.^2^ Joubert’s syndrome is a rare autosomal recessive condition characterized by partial or complete absence of the cerebellar vermis, leading to breathing abnormalities, jerky eye movements, hypotonia, ataxia, impaired equilibrium, and mental handicap.^1^ The term “Joubert syndrome related disorders” (JSRD) was introduced to refer to a group of pleiotropic conditions presenting with the pathognomonic features of JS associated with variable involvement of other organs and systems. These disorders have been classified as ciliopathies.^3,4^

The hallmark imaging features of JS are:

- Dysgenesis of the isthmus (part of the brain stem between the pons and inferior colliculus), which is seen as elongation and thinning of the pontomesen-cephalic junction, and deep interpeduncular fossa.
- Thickening of the superior cerebellar peduncles.

- Hypoplasia of the vermis characterized by incomplete lobulation and enlarged 4th ventricles.
- Incomplete fusion of the halves of the vermis, creating a sagittal vermis cleft seen on axial or coronal MRI planes.

Combination of the first three features produces the characteristic “MTS” on axial MRI.^5-7^ Although MTS is typical of JS, there are other causes of this radiological appearance as well, such as Senior-Loken syndrome, COACH syndrome, Dekaban-Arima syndrome, and Varadi-Papp syndrome^8^ and should be considered under differential diagnosis. Hypogenesis of the vermis results in a triangular-shaped mid-4th ventricle and a “bat wing-shaped” superior 4th ventricle.^1^

Other associated brain anomalies include cortical dysplasias, gray matter heterotopias, ventriculomegaly, and corpus callosum agenesis.^9,10^

Clinical features associated with JS are ocular and oculomotor abnormalities, which are common in JS and are helpful in making a diagnosis.^11^ Abnormalities of ocular motility includes nystagmus, which can be horizontal, vertical, and/or torsional and typically has a pendular or sometimes seesaw pattern, and oculomotor apraxia. Nystagmus and oculomotor apraxia are often present at birth and may improve with age. Other common ocular anomalies may include strabismus, ocular colo-boma, severe visual loss, ptosis, pigmentary changes in the fundus, and decreased vestibulo-ocular reflexes.^12^ Typical respiratory abnormalities in JS are represented by short alternate episodes of apnea and hyperpnea or episodic hyperpnea alone, which tends to occur shortly after birth and progressively improves with age, usually disappearing around the 6th month of life. Their severity can range from occasional short-lasting episodes manifesting every few days to extremely frequent (up to several per day) and prolonged attacks of apnea.^2^ Early hypotonia is observed in nearly all JS patients and can be recognized in the neonatal period or in infancy.^4^ Most studies list hypotonia as one of the major findings of the disease. Maria et al^11^ reported that neonatal hypo-tonia was present in all 59 of their cases. Renal disease often occurs in 25% of individuals with JS. Other renal problems that may be present in JS are renal dysplasia and juvenile nephronophthisis, a form of chronic tubu-lointerstitial nephropathy.^2^ Developmental impairment and intellectual disability are usually severe and present across a variety of domains, including behavior and motor, language, and general development. Although ataxia and balance difficulties are nonspecific findings in JS, they represent a frequent finding due to cerebellar vermis hypoplasia.

Saraiva and Baraitser^13^ in 1992 proposed the following criteria for diagnosis:

- Cerebellar vermis hypoplasia
- Developmental delay
  And at least one of the following manifestations:
- Abnormal eye movements
- Abnormal breathing pattern characterized by episodes of hyperapnea and/or periods of apnea noted in the neonatal period, which improves with age.

The patient in this case report had both clinical and radiological patterns of JS. Polydactyly and syndactyly of all the four limbs were also seen in the patient, which is associated with JS.

Evaluation of a child with suspected JS should include MRI scan, retinal examination, renal ultrasonography, electroretinogram, and karyotyping.^9^ Biallelic mutations in 20 genes, all encoding for proteins of the primary cilium, compose the genetic spectrum of JSRDs. About 50% of the patients carry mutations in the CEP290 gene.^2^ Recent studies have concluded that it is a genetically heterogeneous disorder with one locus mapping to chromosome 9q.^10,14^ A fetal MRI scan between 20 and 22 weeks of gestation has been shown to be an effective method of antenatal diagnosis.^15^

## WHY THIS CASE REPORT IS IMPORTANT TO PEDIATRIC DENTIST

- Emphasis should be placed upon early referral to the pediatric dentist when such a genetic disorder is diagnosed, as they are high-risk patients for dental caries and periodontal diseases. Initiating primary preventive strategies at an early stage helps minimizing the detrimental effects on the oral and perioral structures of the patient.
- The role of pediatric dentist in the management of patients with JS is to provide meticulous preventive regimen like oral prophylaxis for such patients as they lack manual dexterity to perform toothbrushing and have poor oral hygiene status.
- Oral hygiene counseling should be provided by the pediatric dentist to the parents/caretakers of the patients with JS at regular intervals. Parents should be emphasized about the importance of home care to prevent the irreversible adverse sequelae on the child’s oral health at a later stage.
- The pediatric dentist should deal with the cognitive and behavioral abnormalities present in these patients through appropriate behavior management strategies and should also provide the parent with supportive counseling for the rehabilitation of the patient.
- The treatment for such patients is usually supportive. Although treatment can be provided under general anesthesia, not all patients are good candidates for general anesthesia, as JS is associated with airway abnormalities. The presence of high arched palate, micrognathia, and large protruding tongue may further cause difficulty in endotracheal intubation in such patients.
- Medications should be administered with caution, and in consent with the physician, as these patients are sensitive to drugs, which have respiratory depressant effect like opiates and nitrous oxide.
- In patients with associated cleft lip and palate, the pediatric dentist forms an integral part of the multi-disciplinary team responsible for the rehabilitation of the patient.
